# Association between sleep quality and domains of quality of life amongst patients with first episode psychosis

**DOI:** 10.1186/s12955-020-01367-3

**Published:** 2020-04-29

**Authors:** Wei Jie Ong, Xiao Wei Tan, Shazana Shahwan, Pratika Satghare, Laxman Cetty, Boon Tat Ng, Charmaine Tang, Swapna Verma, Siow Ann Chong, Mythily Subramaniam

**Affiliations:** 1 Research Division, Institute of Mental Health, Buangkok Green Medical Park, 10 Buangkok View, Singapore, 539747 Singapore; 2 Pharmancy Department, Institute of Mental Health, Buangkok Green Medical Park, 10 Buangkok View, Singapore, 539747 Singapore; 3 Department of Early Psychosis Intervention, Institute of Mental Health, Buangkok Green Medical Park, 10 Buangkok View, Singapore, 539747 Singapore

**Keywords:** Sleep, Sleep quality, Quality of life, First episode psychosis, Singapore

## Abstract

**Background:**

There is a lack of studies exploring associations between sleep and quality of life (QOL) among patients with schizophrenia who have limited exposure to antipsychotics and are in the early stage of their illness. Our study investigates the association of poor sleep quality and its components with domains of QOL amongst patients with first episode psychosis (FEP).

**Methods:**

Data was utilized from a longitudinal study that examined sleep, smoking and alcohol use amongst patients with FEP who were enrolled in the Early Psychosis Intervention Programme (EPIP). The data were collected during the patients’ baseline visit; i.e., within 3 months of admission into the EPIP. The Pittsburgh Sleep Quality Index (PSQI) was employed to examine sleep quality and its 7 components over the last month. The WHO quality of life-BREF was used to examine QOL and its 4 domains: physical health, psychological, social relationship, and environment. Clinical data such as Positive and Negative Syndrome Scale (PANSS) and Global Assessment of Functioning (GAF) scores were obtained from a clinical data base. Linear regression analyses were conducted to investigate the association between poor sleep quality and the domains of QOL.

**Results:**

Amongst the 280 recruited patients, 62.9% suffered from poor sleep quality. Poor sleep quality was associated with significantly lower scores in all domains of QOL, despite controlling for socio-demographics and clinical variables. Respondents with higher scores in subjective sleep quality and daytime dysfunction were associated with lower scores in the physical health and social relationship domain. Furthermore, respondents with higher scores in subjective sleep quality, sleep latency and daytime dysfunction were associated with lower scores in the psychological domain of QOL. Finally, respondents with higher scores in subjective sleep quality were associated with lower scores in the environment domain of QOL.

**Conclusions:**

Our findings highlight the importance of monitoring sleep quality amongst patients with FEP to improve their QOL. Clinical programmes should also pay more attention to sleep components in order to maintain satisfactory QOL amongst patients with FEP. Future interventions should focus on improving the relevant sleep components to ensure better treatment outcomes.

## Introduction

Schizophrenia is a disabling psychiatric illness that is characterized by symptoms such as hallucinations and delusions, and disruption of thinking for an extensive period of time [1]. The World Health Organization ranked schizophrenia as one of the top 20 leading causes of disability, among other conditions such as loss of hearing, depression and macular degeneration [2]. It is well-established that patients with schizophrenia are likely to suffered from poorer quality of life (QOL) [3, 4].

QOL is defined as the subjective self-evaluation of a person’s current state of life in relation to his or her self-constructed expectations and standards [5]. Many QOL assessment tools, including disease-specific variations, have been developed and validated over the years. Primarily, these tools assess QOL as a multidimensional construct, inclusive of physical, psychological and social functioning [6, 7]. With the emphasis on a more holistic approach towards patient’s care, QOL has slowly gained recognition in mental health services to address the psychosocial aspect of psychiatric illnesses [7–9]. A systematic review identified QOL as a recurring theme in the recovery from psychiatric illness. After synthesizing models of recovery from 15 studies, improving QOL was mapped onto the maintenance and growth stage of the Transtheoretical Model (TTM) of behavior change which focuses on monitoring the continuing change, building resilience and preventing relapse [10]. Hence, in conjunction with monitoring of symptoms, it is necessary to incorporate measurement of QOL in both clinical practice and research to improve the quality of care received by the patients [7, 8]. While QOL measures are used extensively in clinical service and research, there is still a need to further examine the underlying factors associated with the various dimensions of QOL among patients with psychiatric illnesses.

Sleep quality is a complex construct that comprises multiple components. It has been shown to be associated with QOL amongst both the general public and certain clinical populations such as lung cancer survivors and patients with multiple sclerosis [11–13]. Poor sleep quality is a common symptom as well as a consequence of psychiatric illnesses [14]. Like other psychiatric illnesses, patients with schizophrenia often suffer from sleep difficulties [15]. A scoping review has found that poor sleep quality can lead to detrimental impacts on physical health, work performance, cognitive abilities, mental wellbeing and social relationships which are determinants of QOL [16–19].

Previous studies have investigated the relationship between sleep quality and QOL amongst patients with schizophrenia [20, 21]. In these studies, sleep quality was established to be independently associated with the overall and individual subdomains of QOL after controlling for socio-demographic and clinical variables. Moreover, Ritsner et al., highlighted associations between two components of sleep quality (i.e. subjective sleep quality and daytime dysfunction) and overall QOL amongst this population. This finding indicates the possibility that specific components of sleep quality are related to QOL. However, this study was conducted amongst patients with chronic schizophrenia who were prescribed antipsychotics regularly. Furthermore, sleep problems are highly prevalent in the initial phase of the illness, even before the onset of psychotic symptoms [15]. To the best of our knowledge, there is a lack of studies investigating associations between sleep and QOL among populations who have limited exposure to antipsychotics and are in the early stage of their illness.

First episode psychosis (FEP) is usually perceived as the initial stage of developing a psychotic disorder [22]. Compared to patients with chronic schizophrenia in a hospital setting, patients admitted with FEP have limited exposure to the sedative effect of medications, antipsychotics in particular [23]. Hence, the aim of our present study was to explore the relationship between sleep quality and QOL amongst patients with FEP. Specifically, we aimed to examine the association between components of sleep quality and the various domains of QOL. Furthermore, we also explored the association between types of medications used and sleep quality and its components in this population.

## Methods

### Sample

Data was collected from a longitudinal study that examined sleep, smoking and alcohol use amongst patients with FEP who were seeking treatment at the Institute of Mental Health, a tertiary psychiatric hospital or its satellite clinics. A total of 280 outpatients with FEP were recruited for the study. All participants in the study were enrolled in the Early Psychosis Intervention Programme (EPIP); a patient-centered care programme that aims to provide clinical management for patients with FEP for 2 to 3 years. EPIP is led by a multidisciplinary team comprising of psychiatrists, psychologists, case managers, social workers, occupational therapists, nurses, pharmacists and peer support specialists [24]. Patients enrolled in the programme are aged between 16 and 40 years old, diagnosed with a first episode psychotic disorder that was not substance-induced, had been taking antipsychotic medication for duration of less than 12 weeks, and no history of major medical or neurological illness. Only those who were able to read and understand English and were identified as clinically stable by their treating clinicians were included in the current study. In this study, patients were classified as clinically stable if they were symptomatically well-controlled, receiving the same dose of antipsychotics for at least 2 weeks before the study visit and were physically well enough to take part in the study. The data were collected during the patients’ baseline visit, within 3 months of admission into EPIP, from Feb 2014 to Nov 2016. All patients provided written informed consent before their participation in the study. Parental consent was sought for participants who were less than 21 years of age as they are considered minors according to Singapore laws (i.e. age of majority is 21 years in Singapore). The study obtained ethics approval from the National Healthcare Group Domain Specific Review Board.

### Measures

#### Socio-demographic information

Socio-demographic information such as age, gender, ethnic group, marital status, religion, educational level and current work status were collected with a structured self-report questionnaire. Patients’ smoking habit was also assessed in the questionnaire by asking if they had ever smoked. Participants were given 4 options (i.e. Yes, No, Social-smoker, and Ex-smoker) simultaneously. They were expected to select the option that they most identified with. Subsequently, participants were then re-categorized into 3 different categories based on their selection. Those who selected ‘Yes’ were categorized as Current smoker, those who selected ‘No’ were categorized as Never smoked and those who selected ‘Social-smoker’ or ‘Ex-smoker’ were categorized as Social and Ex-smoker.

#### Alcohol use disorder identification test (AUDIT)

All patients were screened for hazardous alcohol use with the Alcohol Use Disorder Identification Test (AUDIT), a 10 items self-reported questionnaire which assesses domains such as alcohol consumption, drinking behaviors and alcohol-related problems [25]. Patients responded to each item on a 5-point Likert scale, scoring from 0 to 4. For this study, those with a total score of 8 and above were classified as ‘hazardous drinker’ [26].

#### WHO quality of life –BREF (WHOQOL-BREF)

The WHOQOL-BREF was employed to measure QOL. It consists of 26 items which covers four different domains: physical health, psychological health, social relationship, and environment [27]. Each item is scored on a 5-point ordinal scale and a mean score is derived for each domain. To transform each domain score into a scaled score, the mean score for each domain was multiplied by 4. Higher scores indicate higher self-perceived QOL. The psychometric properties of WHOQOL-BREF have been validated with a Singapore population comprising of adults with disabilities, adults recovering from mental health issues and adults from the general population [28].

#### The Pittsburgh sleep quality index (PSQI)

The Pittsburgh Sleep Quality Index (PSQI) is a self-reported scale commonly used in clinical research settings. It was employed to examine sleep quality over the last month [29]. It consists of 19 items which cover seven components: subjective sleep quality, sleep latency, sleep duration, habitual sleep efficiency, sleep disturbances, use of sleeping medication, and daytime dysfunction. Following the scoring instruction, individual scores ranging from 0 to 3 were derived for each component with “0” indicating no difficulty and “3” indicating severe difficulty. An overall score is obtained by adding together all seven PSQI components scores with a minimum and maximum score of 0 and 21 respectively. Higher scores indicate poorer sleep quality. Also, a PSQI total score of > 5 is indicative of poor sleep [29].

#### Clinical assessments and medications

As part of the EPIP’s procedure, patients are clinically assessed at baseline and during follow up to monitor clinical outcomes [24]. The Structured Clinical Interview for Diagnostic Statistic Manual of Mental Disorders-IV (SCID-Clinical version) was used to diagnose patients in EPIP at baseline [30]. The duration of untreated psychosis (DUP) was determined as the time, in months, between the onset of psychotic symptoms and the time when the patient was formally diagnosed and started receiving treatment. Positive and Negative Syndrome Scale (PANSS) [31] and Global Assessment of Functioning (GAF) [32] were employed to assess severity of symptoms and functioning respectively. These instruments were administered by well-trained clinicians [24]. Data regarding the clinical assessments and medication (i.e. types of medication use and daily dose of anti-psychotics) was obtained from a clinical database. As majority of the patients were on first generation antipsychotics (FGAs), second generation antipsychotics (SGAs), anti-depressants/mood stabilizers (AD/MS) or any combination of the three groups of medications, data on types of medication use were recoded into 3 individual dichotomous variables: whether the patients were taking 1) FGAs, 2) SGAs, and 3) AD/MS. To standardize the dose of different antipsychotic medications into a single unit for comparison, data on the daily dose of antipsychotics was converted into chlorpromazine (CPZ) equivalents [33–35].

### Statistical analysis

Descriptive statistics were calculated to describe the socio-demographic and clinical characteristics of the sample. Mean and standard deviation were derived for continuous variables while frequency and percentage were derived for categorical variables. Four linear regression analyses were conducted to investigate the association between presence of poor sleep quality and the four individual domains of QOL. To assess the association between the seven PSQI components and the individual domains of QOL, four linear regression analyses were carried out with all components of PSQI as independent variables and each domain of QOL as dependent variable respectively. To assess the association between types of medication use and presence of poor sleep quality, a logistic regression was performed with the 3 groups of medications as independent variables and presence of poor sleep quality as dependent variable. All 3 groups of medications were included in the regression model simultaneously to control for confounding. In a similar manner, a total of 7 linear regression analyses were conducted to explore the association between types of medications used and each of the components of sleep quality separately. Socio-demographic (i.e. age, gender, ethnicity, marital status, religion, educational level, work status, smoking and drinking habits) and clinical variables (i.e. SCID diagnosis, three subscales scores of PANSS, GAF score, estimated CPZ-equivalent dosage and DUP) were included as controls in all regression analyses. SCID diagnoses were also collapsed into 2 broad categories, “Schizophrenia and related psychosis” and “Mood disorder with psychotic symptoms”. “Schizophrenia and related psychosis” included schizophrenia spectrum disorders, delusional disorder, brief psychotic disorder and psychosis not otherwise specific, while “Mood disorder with psychotic symptoms” comprised depression and bipolar disorder with psychotic features. Statistical significance was determined at *p* < 0.05 with two-way tests. All statistical analyses were conducted with IBM SPSS Statistics for windows, version 23.0.

## Results

### Descriptive statistics

Descriptive statistics on the sample’s socio-demographic and clinical characteristics are presented in Table 1. Amongst the 280 recruited patients, the majority were male (50.7%), of Chinese ethnicity (71.4%), never married (85.4%) and diagnosed with schizophrenia and related psychosis (75.7%). Slightly more than half of the patients included in the study had never smoked (59.6%) and only a small proportion of them were classified as having hazardous alcohol use (12.9%). The percentages of patients who were on FGAs, SGAs and the combination of the two were 7.5%, 80.7% and 1.8% respectively. 10% of the patients were not on any antipsychotic. Less than half of the patients were taking AD/MS (31.4%). 27.1% of the patients were on both antipsychotics (i.e. FGAs, SGAs or both) and AD/MS. Majority of the patients suffered from poor sleep quality (62.9%).

**Table 1:** Socio-demographic and clinical characteristics of the sample

		Frequency, N	Percent
Age	20 and below	69	24.6
	21–30	142	50.7
	31–40	69	24.6
Gender	Male	142	50.7
	Female	138	49.3
Ethnic Group	Chinese	200	71.4
	Malay	41	14.6
	Indian	25	8.9
	Other	14	5.0
Marital Status	Ever Married	40	14.3
	Never Married	239	85.4
Religion	Christianity	70	25.0
	Buddhism	74	26.4
	Hinduism	14	5.0
	Islam	56	20.0
	Taoism	6	2.1
	Others	60	21.4
Education	‘O’/‘N’ Level and Below	77	27.5
	‘A’ Level/NITEC/Higher NITEC/Polytechnic Diploma/Other Diploma & Professional Qualification	152	54.3
	University	51	18.2
Work Status	Student/Homemaker/Housewife	78	27.9
	Working/National Service	118	42.1
	Unemployed	78	27.9
SCID Diagnosis	Schizophrenia and related psychosis	212	75.7
	Mood disorder with psychotic symptoms	23	8.2
Smoking Habit	Never smoked	167	59.6
	Current smoker	95	33.9
	Social and ex-smoker	17	6.1
AUDIT score	No-hazardous use (score < 8)	244	87.1
	Hazardous use (score ≥ 8)	36	12.9
PSQI score	Poor sleep quality (score > 5)	176	62.9
	Normal sleep quality (score ≤ 5)	98	35.0
Taking FGAs	Yes	26	9.3
	No	254	90.7
Taking SGAs	Yes	231	82.5
	No	49	17.5
Taking AD/MS	Yes	88	31.4
	No	192	68.6
		Mean	SD
Baseline PANSS	Positive	21.89	5.994
	Negative	15.76	8.733
	GPS	38.16	11.346
Baseline GAF	Total	44.30	12.062
DUP since onset of symptoms (in months)		13.55	21.685
Daily dosage converted to CPZ		279.0325	216.7946
PSQI	Component 1: Subjective Sleep Quality	1.11	0.839
	Component 2: Sleep Latency	1.45	0.995
	Component 3: Sleep Duration	0.81	1.119
	Component 4: Habitual Sleep Efficiency	0.69	1.040
	Component 5: Sleep Disturbances	1.34	0.751
	Component 6: Use of Sleeping Medication	1.14	1.316
	Component 7: Daytime Dysfunction	1.24	0.972
	Global PSQI Score	7.81	4.429
WHOQOL-BREF	Physical health	13.89	2.742
	Psychological	12.13	3.148
	Social Relationship	12.83	3.058
	Environment	13.40	2.775

Note: 1 missing response for marital status (0.4%); 6 missing response for work status (2.1%); 45 missing response for SCID diagnosis (16.1%); 1 missing response for smoking habit (0.4%); 6 missing response PSQI (2.1%)

*AD* anti-depressant, *AUDIT* alcohol use disorder identification test, *CPZ* chlorpromazine, *DUP* duration of untreated psychosis, *FGAs* first generation antipsychotics, *GAF* general assessment of functioning, *GPS* general psychopathology, *MS* Mood Stabilizer, *PANSS* positive and negative syndrome scale, *PSQI* Pittsburg sleep quality index, *SCID* structured clinical interview for diagnostic and statistical manual of mental diseases, *SGAs* Second generation antipsychotics, *WHOQOL-BREF* World Health Organization Quality of Life - BREF

### Association between sleep quality and QOL

Poor sleep quality was associated with significantly lower scores in all subdomains of QOL after controlling for socio-demographics and clinical variables (Table 2).

**Table 2:** Association between poor sleep quality and domains of WHOQOL-BREF

Domains of WHOQOL-BREF	B	Standard Error	*P* value	95% Confidence Interval	
				Lower Bound	Upper Bound
Physical health	−2.236	0.346	< 0.001*	−2.919	−1.554
Psychological	−1.967	0.431	< 0.001*	−2.817	−1.118
Social Relationship	−1.376	0.451	0.003*	−2.266	−0.486
Environment	−1.655	0.357	< 0.001*	−2.359	−0.950

*PSQI* Pittsburg sleep quality index, *WHOQOL-BREF* World Health Organization Quality of Life - BREF

*P value < 0.05

Similarly, respondents with higher scores in subjective sleep quality and daytime dysfunction were associated with lower scores in the physical health (Table 3) and social relationship domains of QOL (Table 4). Respondents with higher scores in subjective sleep quality, sleep latency and daytime dysfunction were associated with lower scores in the psychological domain of QOL (Table 5). Finally, respondents with higher scores in subjective sleep quality were associated with lower scores in the environment domain of QOL (Table 6).

**Table 3:** Association between each component of PSQI and physical health domain of WHOQOL-BREF

Component of PSQI	B	Standard Error	*P* value	95% Confidence Interval	
				Lower Bound	Upper Bound
Subjective sleep quality	− 0.913	0.220	< 0.001*	− 1.348	- 0.479
Sleep latency	− 0.165	0.164	0.315	− 0.489	0.158
Sleep duration	−0.151	0.161	0.351	−0.469	0.167
Habitual sleep efficiency	0.121	0.158	0.445	−0.191	0.433
Sleep disturbances	−0.060	0.221	0.787	−0.497	0.377
Use of sleeping medicine	−0.076	0.116	0.513	−0.305	0.153
Daytime Dysfunction	−0.863	0.177	< 0.001*	−1.212	− 0.513

*PSQI* Pittsburg sleep quality index, *WHOQOL-BREF* World Health Organization Quality of Life - BREF

*P value < 0.05

**Table 4:** Association between each component of PSQI and social relationship domain of WHOQOL-BREF

Component of PSQI	B	Standard Error	*P* value	95% Confidence Interval	
				Lower Bound	Upper Bound
Subjective sleep quality	−0.623	0.308	0.045*	−1.231	−0.015
Sleep latency	−0.365	0.230	0.114	−0.818	0.088
Sleep duration	−0.274	0.226	0.744	−0.519	0.371
Habitual sleep efficiency	−0.091	0.221	0.682	−0.528	0.346
Sleep disturbances	−0.138	0.310	0.657	−0.749	0.473
Use of sleeping medicine	0.195	0.163	0.232	−0.126	0.516
Daytime Dysfunction	−0.649	0.248	0.010*	−1.138	−0.160

*PSQI* Pittsburg sleep quality index, *WHOQOL-BREF* World Health Organization Quality of Life - BREF

*P value < 0.05

**Table 5:** Association between each component of PSQI and psychological domain of WHOQOL-BREF

Component of PSQI	B	Standard Error	*P* value	95% Confidence Interval	
				Lower Bound	Upper Bound
Subjective sleep quality	−0.632	0.294	0.033*	−1.212	−0.052
Sleep latency	−0.633	0.219	0.004*	−1.065	−0.201
Sleep duration	−0.287	0.215	0.184	−0.711	0.138
Habitual sleep efficiency	0.048	0.211	0.822	−0.369	0.464
Sleep disturbances	0.504	0.295	0.090	−0.079	1.086
Use of sleeping medicine	0.010	0.155	0.948	−0.296	0.316
Daytime Dysfunction	−0.694	0.236	0.004*	−1.160	−0.227

PSQI – Pittsburg sleep quality index; WHOQOL-BREF – World Health Organization Quality of Life - BREF

*P value < 0.05

**Table 6:** Association between each component of PSQI and environment domain of WHOQOL-BREF

Component of PSQI	B	Standard Error	*P* value	95% Confidence Interval	
				Lower Bound	Upper Bound
Subjective sleep quality	−0.517	0.251	0.040*	−1.012	−0.023
Sleep latency	−0.308	0.187	0.100	−0.677	0.060
Sleep duration	−0.175	0.183	0.341	−0.537	0.187
Habitual sleep efficiency	−0.233	0.180	0.198	−0.588	0.123
Sleep disturbances	−0.189	0.252	0.455	−0.685	0.308
Use of sleeping medicine	−0.037	0.132	0.779	−0.298	0.223
Daytime Dysfunction	−0.296	0.202	0.144	−0.694	0.102

*PSQI* Pittsburg sleep quality index, *WHOQOL-BREF* World Health Organization Quality of Life - BREF

*P value < 0.05

### Association between types of medications used and sleep quality

Taking FGAs or SGAs was not associated with sleep quality and any of the sleep quality components (data available on request). Although not statistically significant, there is a trend towards poor sleep quality amongst patients who were on AD/MS (*P* = 0.05). Furthermore, patients who were on AD/MS were significantly associated with higher scores in sleep latency and daytime dysfunction (Table 7).

**Table 7:** Association between taking AD/MS and components of sleep quality

Medication	Sleep latency					Daytime dysfunction				
	B	SE	*P* value	95% CI		B	SE	*P* value	95% CI	
				Lower	Upper				Lower	Upper
AD/MS	0.364	0.169	0.033*	0.030	0.698	0.591	0.161	< 0.001*	0.273	0.909

*AD* anti-depressant, *MS* mood stabilizer

*P value < 0.05

## Discussion

Amongst the 280 patients with FEP who participated in the study, 62.9% were identified as having poor sleep quality. This rate is higher than the prevalence of poor sleep quality amongst patients with chronic schizophrenia who were under regular treatment with antipsychotics (i.e. 45.4%) as reported by Ritsner, Kurs [20]. It is possible that this difference is due to limited exposure to the sedative effects of antipsychotics amongst our participants with FEP who are also less likely to be receiving polypharmacy with the additive sedative effect. Moreover, our study found that those participants with poor sleep quality were more likely to have poorer evaluation on all four subdomains of QOL, before and after controlling for socio-demographic and clinical variables. This finding is consistent with other studies suggesting that sleep quality is independently associated with QOL [20, 21].

In our study, three of the seven components of sleep quality were associated with QOL domains amongst patients with FEP, namely subjective sleep quality, daytime dysfunction and sleep latency, which is similar to a previous study among patients with chronic schizophrenia [20]. The finding indicated that those with poorer subjective sleep quality were more likely to report poorer scores in all QOL domains. Semler and Harvey found an association between subjective perception of sleep quality and ratings of daytime functioning amongst patients with insomnia, regardless of their actual sleep [36]. A systematic review on how people with serious mental illness (SMI) such as psychotic illnesses perceived sleep concluded that patients with SMI generally perceived poor sleep quality as one of the most troubling symptoms of their illness [37]. Moreover, the patients also believed that poor sleep quality can lead to impairment in areas such as mood, mental wellbeing, energy, motivation and functioning, factors which correspond to multiple facets assessed in the domains of QOL [27, 37]. Hence, given that poor sleep quality adversely impacts their life, it is possible that those with poorer subjective sleep quality were more likely to overestimate the effect of poor sleep quality, resulting in poorer evaluation of QOL domains.

Participants with more severe daytime dysfunction were more likely to have lower QOL in the physical health, psychological and social relationship domains. This finding is consistent with a similar study which identified a correlation between daytime dysfunction, and both physical and mental health domains of QOL amongst patients with mechanical circulatory support [38]. Severity of daytime dysfunction is determined by the frequency of having difficulties staying awake for daily activities and the degree of difficulty in maintaining motivation to complete everyday tasks [29]. Studies observed that frequent daytime sleepiness is associated with impaired physical functioning, lower frequency of exercise and elevated psychological stress [39, 40]. Furthermore, motivation was also found to be predictive of social activity and cognitive performance [41]. Hence, patients who reported having more severe daytime dysfunction are more likely to experience the associated negative effects on physical health, psychological and social functioning, which leads to poorer evaluation on the domains of QOL. In the present study this association between daytime dysfunction and the 3 domains of QOL is still evident even after adjusting for GAF score which suggests that FEP patients might perceive the impact of sleep-induced dysfunction on QOL independently to some degree from symptoms-induced dysfunction in general. One possibility is that sleep is commonly seen as a basic necessity, hence dysfunction associated with poor sleep quality is seen as highly undesirable. Nevertheless, further research is required to explore this hypothesis.

Those with more difficulties initiating sleep were more likely to report having lower QOL in the psychological domain in the current sample. This finding may be attributed to the association between sleep latency and anxiety. An epidemiological study conducted to investigate the sleep habits and sleep disturbances among elderly subjects in Iceland found that difficulties initiating sleep was associated with higher anxiety [42]. Patients with anxiety disorders also frequently suffer from prolonged sleep latency [43, 44]. Moreover, therapies aimed toward relaxation and reduction of anxiety are also found to be effective on patients with sleep-onset insomnia [45]. Hence, there are two explanations to our observations: 1) prolonged sleep latency amongst patients with FEP may lead to increase anxiety and results in poorer evaluation of the psychological domains of QOL; 2) patients with FEP may report having poorer QOL because of heightened anxiety which leads to difficulties in initiating sleep subsequently. However, future research should examine the association between sleep latency and anxiety specifically amongst patients with FEP and schizophrenia to substantiate these explanations.

Although it is well documented in literature that antipsychotics affect sleep, our study failed to find any significant association between use of antipsychotic (i.e. FGAs and SGAs) and sleep quality and its components (data available on request) [23]. This may be because of the limited exposure to antipsychotics amongst this population, hence its effect on sleep was not as significant. Despite that, our study indicated a trend towards poor sleep quality amongst patients who were on AD/MS. Furthermore, patients who were on AD/MS were more likely to have longer sleep latency and more severe daytime dysfunction. It is also well known that anti-depressants and mood stabilizers affect sleep [46–48]. Although some patients reported improvement with sleep quality, many patients who were on anti-depressants had frequent complaints of insomnia and daytime sleepiness [47, 48]. Similarly, mood stabilizers also possess sedative effect [46]. Moreover, it was suggested in literature that the effect of anti-depressants on sleep are the strongest during the initial few weeks of treatment [48]. This would explain the association found between use of AD/MD and the relevant sleep components amongst FEP patients early in their treatment.

There are several limitations that should be taken into consideration while interpreting the results of the study. Firstly, our study is cross sectional in nature, hence no causal relationship can be concluded from the findings. Secondly, there are issues pertaining to the use of self-reported measures. Both our variables of interest, sleep quality and QOL were assessed with self-administered measures, therefore the data collected for these variables are subjected to possible social desirability bias. Thirdly, we did not collect data on caffeine consumption which may have a possible effect on sleep quality. Finally, although studies have found that depression and anxiety are associated with both sleep quality and QOL [17, 42, 49], our study did not include specific measures to assess comorbid depression and anxiety conditions.

## Conclusion

In conclusion, our finding emphasizes the importance of monitoring sleep quality amongst patients with FEP to better improve their QOL. Our study also highlights subjective sleep quality, daytime dysfunction and sleep latency as components of sleep quality associated with various aspects of QOL. Hence, future interventions should focus on improving these sleep components among patients with FEP to ensure better treatment prospects, especially in terms of improving subjective QOL. Other than pharmacological interventions, non-pharmacological interventions such as cognitive behavioral therapy, relaxation therapy and daytime bright light therapy should also be included as treatment to improve sleep quality amongst this population [50, 51]. Furthermore, it is important for clinicians to prescribe anti-depressants and mood stabilizers cautiously to avoid detrimental effects on the patients' sleep quality.

## Data Availability

The datasets used and/or analysed during the current study are available from the corresponding author on reasonable request.

## Abbreviations

AD: Anti-depressant
AUDIT: Alcohol use disorder identification test;
CPZ: Chlorpromazine
DUP: Duration of untreated psychosis
FGAs: First generation antipsychotics
GAF: General assessment of functioning
GPS: General psychopathology
MS: Mood Stabilizer
PANSS: Positive and negative syndrome scale
PSQI: Pittsburg sleep quality index
SCID: Structured clinical interview for diagnostic and statistical manual of mental diseases
SGAs: Second generation antipsychotics
WHOQOL-BREF: World Health Organization Quality of Life – BREF
